# The effectiveness and safety of nonsurgical integrative interventions for symptomatic lumbar spinal spondylolisthesis: A randomized controlled multinational, multicenter trial protocol

**DOI:** 10.1097/MD.0000000000010667

**Published:** 2018-05-11

**Authors:** Kiok Kim, Yousuk Youn, Sang Ho Lee, Jung Chul Choi, Jae Eun Jung, Jaehong Kim, Wenchun Qu, Jason Eldrige, Tae-Hun Kim

**Affiliations:** a Department of Spine Center, Mokhuri Neck and Back Hospital, Seoul; b Hongik Neurosurgery Hospital, Seongnam, Republic of Korea; c Department of Physical Medicine and Rehabilitation; d Department of Anesthesiology, Division of Pain Medicine, Mayo Clinic, Rochester, MN, USA; e Korean Medicine Clinical Trial Center, Korean Medicine Hospital, Kyung Hee University, Seoul, Republic of Korea.

**Keywords:** acupuncture, Chuna, clinical trial, conventional therapy, epidural steroid injection, non-surgical treatment, protocol, Spondylolisthesis

## Abstract

**Background:**

Surgery is generally accepted as the main therapeutic option for symptomatic lumbar spondylolisthesis. However, new nonsurgical therapeutic options need to be explored for this population.

**Objectives:**

The objective of this study is to assess the effectiveness and safety of a 5-week Mokhuri treatment program compared with conventional nonsurgical treatments for symptomatic lumbar spondylolisthesis.

**Methods:**

This is a study protocol for a multinational, multicenter clinical randomized controlled trial comparing the effectiveness and safety of 5 weeks of nonsurgical integrative treatments (a Mokhuri treatment program consisting of Chuna, acupuncture, and patient education) with nonsurgical conventional treatments (drugs for pain relief, epidural steroid injections, and physical therapy). Clinical outcomes including visual analogue scale (VAS) scores ranging from 0 to 100 for low back pain and leg pain, EQ-5D scores, Oswestry disability index (ODI) scores, Roland–Morris Disability Questionnaire (RMDQ) scores, Zurich Claudication Questionnaire (ZCQ) scores, walking duration and distance without leg pain, and a 5-minute treadmill test, and the ratio between the actual duration of participation and the originally scheduled duration in each group, the presence of any additional spondylolisthesis treatments, the types of concomitant treatments during the follow-up period, and adverse events (AEs) will be assessed at 7 weeks, 18 weeks, 30 weeks, 54 weeks, and 102 weeks after the end of the treatments.

**Conclusion and discussion:**

The results of this study will provide clinical evidence on nonsurgical integrative interventions for patients with symptomatic lumbar spondylolisthesis.

**Clinical trial registry:**

clinicaltrials.gov (NCT03107468)

## 1 Introduction

Degenerative lumbar spondylolisthesis is a chronic spinal condition that causes instability of the segmental spinal bones due to degenerative changes in the spinal joints. According to a cross-sectional study released in 2009, the prevalence rate of degenerative lumbar spondylolisthesis was estimated to be as high as 13.6% in an adult community-based population, which suggests that a considerable proportion of general population have this condition.^[1]^ Most people with lumbar spondylolisthesis do not have any symptoms, and only approximately 10% of this population have clinical symptoms that require treatment. However, once symptoms occur, more than 50% of the patients complain of considerable pain and dysfunction and in severe cases, cauda equina syndrome.^[2]^

Nonsurgical conservative treatments are recommended for patients with symptomatic spondylolisthesis as a first-line therapy, but surgical treatments are considered in cases of failure of conservative treatments.^[3]^ Surgical interventions are performed to achieve decompression of nervous tissue, to relieve joint instability, and to lead to a comparatively good prognosis.^[4]^ However, considerable complication rates related to surgical interventions and considerable reoperation rates after spinal surgery have been reported.^[5,6]^ In addition, surgical treatment should be avoided if the patient has severe chronic conditions, multilevel stenosis, osteoporosis, or poor compliance.^[3]^ Considering this, nonsurgical interventions are recognized as an important option for the management of lumbar spinal spondylolisthesis.

According to the results of our previous observational study involving Mokhuri nonsurgical integrative treatments performed on patients diagnosed with Meyerding grade 2 lumbar spinal spondylolisthesis, considerable clinical improvement was observed after nonsurgical integrative treatments; the average walking distance without pain increased from 55 to 165 m, while the intensity of pain experienced when walking was reduced by 80% compared with the baseline assessment.^[7]^ In another previous retrospective audit study, on a numeric rating scale from 0 to 10, 44 spondylolisthesis patients reported a reduction in numeric rating scale scores from 7.1 to 3.1, and walking distance without pain increased from 193 to 568 m after treatment.^[8]^ These observational studies provide clues for the potential benefit of nonsurgical integrative treatments and suggest that these treatments might be effective for pain relief and functional restoration in patients with symptomatic lumbar spinal spondylolisthesis. However, observation studies cannot provide clinical evidence, so these new therapeutic options should be evaluated through rigorous, high-powered clinical trials.

The objective of this study is to assess the effectiveness and the safety of a 5-week Mokhuri treatment program compared with conventional nonsurgical treatments for symptomatic lumbar spondylolisthesis.

## 2 Methods

### 2.1 Study design

This is a study protocol for a multinational, multicenter clinical randomized controlled trial that is designed to evaluate the effectiveness and safety of nonsurgical integrative treatment in patients with symptomatic lumbar spinal spondylolisthesis who continue to have symptoms after a sufficient duration of conservative treatment. Patients with symptomatic lumbar spinal spondylolisthesis will be randomly allocated to one of the interventions: a 5-week Mokhuri treatment program (Chuna, acupuncture, and patient education) or nonsurgical conventional standard treatment (drugs for pain relief, epidural steroid injections, and physical therapy). The trial participants who agree to participate in the clinical trial after written informed consent will undergo the required examinations and tests according to the plan for the clinical trial. After 5 weeks of treatment, the clinical outcomes of both groups will be evaluated during follow-up, which will take place at 7, 18, 30, 54, and 102 weeks after the end of the treatments.

### 2.2 Study setting

The trial will be conducted at the Mayo Clinic in the United States and the Mokhuri Oriental Medicine Hospital in Korea. Participants will be recruited at the division of Pain Medicine at the Mayo Clinic in Minnesota, US and at the Mokhuri Oriental Medicine Hospital in Seoul, South Korea. Participants will be recruited through flyer advertisement within the hospital, e-mail alert campaigns, and public advertisement. Any included patients will undergo 5 weeks of treatments in the outpatient unit and up to 96 weeks of follow-up evaluations.

### 2.3 Inclusion and exclusion criteria

Individuals who meet all the following criteria will be included as appropriate participants in the clinical trial:

(1) Those aged from 19 to 78 years;
(2) Those with a diagnosis of degenerative lumbar spinal spondylolisthesis or who have low back pain, lower limb radiating pain, or leg discomfort when standing or walking with a severity of at least 5 on a visual analogue scale (VAS) of 0 to 100 for each symptom;
(3) Those suffering from neurologic claudication or radicular pain for at least 1 year;
(4) Those with neurogenic claudication within 5 minutes of walking on a treadmill at a speed of 1.5 miles per hour;
(5) Those who have not received an epidural injection within the past month;
(6) Those who have not undergone lumbar surgery;
(7) Those who have confirmed spondylolisthesis on lumbar spine AP, lateral, nd both oblique views;
(8) Those who weigh 250 lbs (113.398 kg) or less;
(9) Those whose height is 2.1 m (6.890 ft) or shorter; and
(10) Those who agreeing to participate in this clinical trial after receiving a thorough explanation of the purposes and characteristics of the trial and who are willing to sign the written informed consent form.

Participants who have any of following conditions will be excluded:

(1) Those with a past or current history of diseases that cause ambulatory functional disability;
(2) Those with knee joint and hip joint disorders that severely limit walking (i.e., moderate or severe osteoarthritis of the knee or hip joints);
(3) Those who have been previously diagnosed with peripheral blood vessel diseases or vascular diseases, those who show an ankle-brachial index below 0.9 or those who have been diagnosed with peripheral arterial disease by Doppler ultrasonography of lower limbs, if necessary;
(4) Those with severe diseases (cardiac disorders or renal insufficiency) to such a degree that an ambulatory evaluation cannot be performed;
(5) Those with other specific spinal diseases (ankylosing spondylitis, spinal osteomyelitis, metabolic diseases, severe osteoporosis, etc.);
(6) Those with severe neurological defects including foot drop or cauda equina syndrome;
(7) Those with spinal instability confirmed by lumbar spine X-ray flexion and extension views, where spinal instability will be defined as observing one or more of the following: 4.5 mm sagittal plane translation, 20 degrees of sagittal plane rotation at L4-5, or 25 degrees of sagittal plane rotation at L5-S1;
(8) Those with malignancies;
(9) Those with past or current psychiatric conditions, such as major depressive disorder, anxiety disorders, panic disorders, or episodes of mania, delusion, and schizophrenia;
(10) Those using narcotic analgesics including external dosage forms or patches;
(11) Those women who are pregnant, lactating, or planning to become pregnant;
(12) those who appear to be likely to encounter difficulties in adhering to this protocol; and
(13) Those subjects whom the clinical investigators judge to be inappropriate.

### 2.4 Interventions

All the participants in each group will undergo 5 weeks of treatments. In the Mokhuri treatment group, participants will undergo 10 sessions of acupuncture therapy, Chuna therapy, and consultation with physicians. In the control group, participants will be administered drugs every day, have 1 or 2 epidural steroid injections, and participate in 10 sessions of physical therapy for 5 weeks.

### 2.5 Interventions in the Mokhuri treatment group

All the treatments in this group will be conducted by Korean Medical doctors in Korea and an acupuncture practitioner in the US, both with at least 5 years of clinical practice experience. All the treatments in this group will be offered twice a week for 5 weeks (a total of 10 sessions). For acupuncture treatment, points including both LI4, ST36, LV3, BL22, BL23, BL24, and BL25 will be selected for stimulation. Disposable sterile acupuncture needles (0.25 × 40 mm, Dong-bang Acupuncture, Seong-Nam, Korea) will be used. Chuna treatment, which is a kind of manual therapy consisting of relaxation and mobilization of the lumbar joint sand back muscles using an Ergo Style TM FX-5820 Table (Chattanooga Group, TN), will be conducted during every treatment visit. Participants will lie on the table in the prone position, and a practitioner will relax the back muscles of the patient, while the table causes the patient's spine to flex and extend automatically.^[9]^ Along with the acupuncture and Chuna treatment, patient consultation regarding advice for everyday life activities, walking, and particular movements and exercises will be offered to participants during every visit.

### 2.6 Interventions in the control group

Participants in the control group will undergo 5 weeks of nonsurgical conventional standard treatments including drug therapy, epidural injections, and physical therapy. Through consultation with neurosurgeons or anesthesiologists or rehabilitation medicine specialists, drugs including muscle relaxants, NSAIDs, gabapentin, pregabalin or tricyclic antidepressants will be prescribed based on each patient's condition. Epidural steroid injections will be performed once or twice during the 5-week participation period. Physicians will assess the participants’ pain condition and decide whether they might need an epidural steroid injection at each visit. Physicians will re-evaluate the pain severity of the participant and perform epidural injections after a 2-week interval if necessary. Epidural injections will be performed at the affected spinal level. Physiotherapists will conduct physical therapies, including heating pad application for 10 to 20 minutes and transcutaneous electrical nerve stimulation for 5 to 10 minutes, twice a week during the 5 weeks of participation.

### 2.7 Allowed cotreatments

According to the investigators’ decisions, medications that do not appear to influence the interpretation of the results of this clinical trial, such as medicines the participants have been taking for 4 weeks prior to participating, will be allowed. In addition, in both the Mokhuri treatment group and the conventional treatment group, participants will be allowed to continue using the same types of pain medications at the same doses and frequencies that they have been taking them before participating in this study.

### 2.8 Research personnel training

One month of training for practitioners participating in the treatments will be planned at 5 days a week for 8 hours a day for 160 hours in total. The training program will include the theory of the treatments, the practice of Chuna, and patient education. A common standard operating procedure (SOP) for treatments will be provided, and practitioners will follow this SOP. At the end of every week, trainees will be evaluated for competence.

### 2.9 Sequence generation and allocation concealment

Two sets of random sequences will be generated for the 2 research institutions using the statistical program SAS (SAS institute Inc, Cary, NC) by an independent statistician. The block size will not be known to the investigators so as not to predict the random sequence. All participants will have equal chances of being selected for either the Mokhuri treatment group or the control group. Sealed opaque envelopes with the assigned results for each participant will be used for allocation concealment. The investigators at each of the study institutions will open a random assignment envelope and assign the participant to an intervention group in the consecutive order of the patients’ serial numbers.

### 2.10 Outcomes

The validated English version for the Mayo Clinic and the Korean version for the Mokhuri hospital will be used for all the outcome assessments. Outcome assessors will be blinded to the allocation results.

The primary outcome will be VAS scores from 0 to 100 for low back pain. The degree of pain will be evaluated on a 100 mm straight line, where the left edge of the straight line will indicate “0: no symptoms,” and the right edge will indicate “100: the most severe pain.” The participant will indicate the average level of lumbar pain experienced for the past week.

The secondary outcomes will include VAS scores from 0 to 100 for leg pain, EQ-5D scores, Oswestry disability index (ODI) scores, Roland–Morris Disability Questionnaire (RMDQ) scores, Zurich Claudication Questionnaire (ZCQ) scores, walking duration and distance without leg pain, a 5-minute treadmill test, the ratio between the actual duration of participation and the originally scheduled duration in each group, the presence of any additional spondylolisthesis treatments, the types of concomitant treatments during the follow-up period, and adverse events (AEs).

For assessing leg pain, a VAS score from 0 to 100 for leg pain will be assessed. The participant will indicate the average level of leg pain experienced for the past week. The EQ-5D is a common assessment tool for measuring health-related quality of life. It contains 5 dimensions including mobility, self-care, usual activities, pain (discomfort), and anxiety (depression). In this study, we will use the EQ-5D-3L, which has 3 levels of severity for each dimension.^[10,11]^ The ODI consists of 10 topics concerning pain, lifting, ability of self-care, ability to walk, sit, stand and travel, sexual function, social life, and sleep quality and is intended to assess disability and quality of life related to low back pain. ODI scores range from 0 (no disability) to 100 (maximum disability possible).^[12,13]^ The RMDQ is a tool that is widely used for evaluating the functional conditions of patients with low back pain. It contains 24 statements regarding the patient's perceptions about pain and disability, such as physical activity, sleep, psychosocial, and pain frequency. RMDQ scores range from 0 (no disability) to 24 (maximal disability possible).^[14,15]^ The ZCQ is an instrument for assessing pain and symptoms related to spinal stenosis. It consists of 3 domains with 18 questions assessing the symptom severity over the last month, physical functioning over the last month on a specific day, and the patient's satisfaction with current treatment. The results are recorded as a percentage of the possible maximum score, and higher scores represent worse conditions.^[16]^ The walking duration and distance without leg pain and a treadmill test will be used to assess the physical function of the patients. Patients will be asked to walk a distance of 25 m on a round track at their preferred speed, and the distance will be measured when they are no longer able to walk due to pain in the lower limb or lumbar region. For the treadmill test, the participants will walk on the level treadmill at a speed of 1.5 miles per hour, and the time until they start to feel pain in their legs will be measured.^[17]^ The presence of any additional treatments for spondylolisthesis and the types of treatments will be assessed during the follow-up period. Because the treatments in each of the groups will end, patients will be allowed to use additional treatments for spondylolisthesis if they wish to. During this period, participants will be asked whether they used other additional treatments for symptoms related to spinal spondylolisthesis and what types of treatments they used. The ratio between the actual duration of participation and the originally scheduled duration in each group will be assessed as well. Participants whose VAS scores from 0 to 100 for back pain and leg pain decrease below 1 at every weekly evaluation will stop the treatment immediately, according to the early termination rule of this study. The number of patients who stop the scheduled treatments early and the number of those who finish all the scheduled treatments will be assessed in each group, and the differences in the ratio between groups will be assessed. AEs will be assessed during each visit. Participants will be asked to report AEs voluntarily, and researchers will observe participants’ conditions on a regular basis. For each reported AE, the symptoms, signs, starting date, duration, severity, and causal relationship will be assessed by researchers.

### 2.11 Participant time schedule

At the screening visit, participants will be given detailed explanations of the objectives and contents of this clinical trial and will receive the written agreement form. During this visit, a 5-minute treadmill test, lumbar physical and radiological examinations, ankle-brachial index assessment (or Doppler ultrasonography of lower limbs if necessary), bone mineral densitometer assessment, and inclusion criteria assessment will be conducted. If the participant can be enrolled in the study, random assignment of the participant will take place at the following visit. Before the treatments begin, the VAS scores for low back pain and for leg pain, EQ-5D scores, ODI scores, RMDQ scores, ZCQ scores, walking duration and distance free of leg pain, and a treadmill test will be assessed. After that, participants will undergo 10 treatments over 5 weeks. After treatment, evaluations will be performed immediately (7 weeks) and at 18 weeks, 30 weeks, 54 weeks (1-year follow-up), and 102 weeks (2-year follow-up). AEs will be assessed at each visit (Table 1).

**Table 1:**
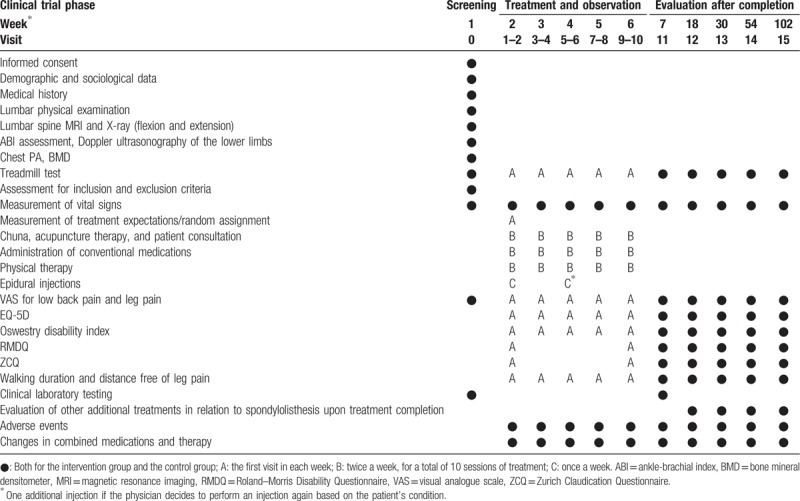
Participant schedule.

### 2.12 Sample size

To calculate the sample size, a retrospective study performed on 44 patients with lumbar spinal spondylolisthesis^[8]^ and the previous clinical trial data that assessed physical therapy for hospitalized patients in a similar study setting^[18]^ were used. To determine a meaningful difference between groups, a minimal clinically important difference of 2.5 points on the numeric rating scale of 0 to 10 was used.^[19]^ The standard deviation (SD) was 4.65 from the abovementioned previous studies. For the sample size calculation, the significance level (α) was 0.05, and the power (1–β) was 0.80. The following formula was used for the calculation. Assuming a drop-out rate of 10%, 61 participants will be needed in each study group, yielding a total of 122 participants needed for this study. 
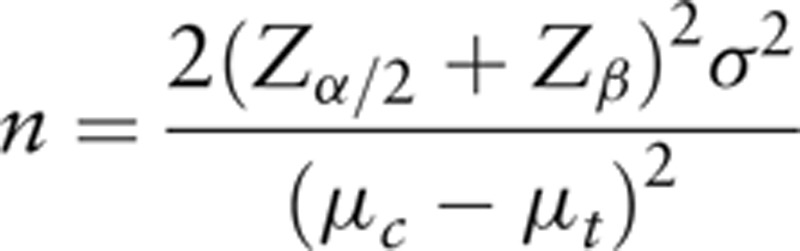


In terms of the total 122 participants in the study, 108 participants will be recruited at the Mokhuri Oriental Medicine Hospital and 14 participants at the Mayo Clinic.

### 2.13 Statistical analysis

Statistical analysis will be performed based on an intention-to-treat analysis for the assessment of effectiveness outcomes and a per-protocol analysis for the assessment of safety. The missing values will be substituted with the most recently observed value (the last observation carried forward method). For the demographic information, continuous data will be presented as the mean and SD, and categorical data will be reported with a frequency table. The 2-sample *t* test (or Wilcoxon rank-sum test) will be used for the analysis of continuous variables after evaluating the data distribution. For categorical data, the chi-square test (or Fisher exact test) will be used. Outcome variables including VAS scores for lumbar pain and leg pain, EQ-5D scores, ODI scores, RMDQ scores, ZCQ scores, walking duration and distance without leg pain, and the treadmill test will be summarized with means and SDs. For statistical analysis of continuous outcomes, changes in the scores before and after treatments will be regarded as dependent variables, with the baseline scores and research institutions as covariates and the groups as fixed factors, to perform the analysis of covariance. To verify the trends for each visit in the 2 groups, repeated measures analysis of variance will be performed. For categorical outcome data, the chi-square test (or Fisher exact test) will be used.

### 2.14 Ethics

Approval from each institutional review board has been obtained for the protocols before the start of this study (institutional review board application number: MHNBH-16031 at the Mokhuri hospital and 15-008457 at the Mayo Clinic). Prior to the clinical trial, the participants will provide written informed consent for participation in the study.

## 3 Discussion

This is a multinational, multicenter study protocol for a clinical trial assessing the effectiveness and safety of nonsurgical integrative interventions for symptomatic lumbar spondylolisthesis. Conservative interventions are regarded as effective in most lumbar spinal spondylolisthesis patients, but there are no appropriate options other than spinal surgery for patients who have continuing symptoms after conservative treatment.^[20]^ As surgery is not always promising for all patients, new nonsurgical therapeutic options need to be explored for this population.

This multinational study will ensure generalizability of the study results. This is the most important strong point of this study. Generalizability is an important issue when implementing the results of studies into clinical practice. An randomized controlled trial, which can reduce the risk of bias in study participant selection, may not be able to ensure external validity if the study population is limited to homogeneous group under strict eligibility criteria.^[21]^ From this perspective, a multinational, multicenter clinical trial has benefits of ensuring generalizability because patients with diverse regional and ethnic characteristics are recruited.

One important limitation of this study is its lack of internal validity. Researchers with different levels of clinical experience and educational backgrounds will participate in this study, so internal validity between the 2 research institutions may be a potential problem. To protect against potential bias from this aspect, we generated a common SOP for treatments and conducted 1 month of an integrated education program for researcher proficiency.

Hopefully, this study will provide clinical evidence on nonsurgical integrative interventions for symptomatic spondylolisthesis.

## Acknowledgment

The authors thank a grant of the Korea Health Technology R&D Project through the Korea Health Industry Development Institute (KHIDI), funded by the Ministry of Health & Welfare, Republic of Korea (grant number: HI16C1625).

## Author contributions

**Conceptualization:** Kiok Kim, Yousuk Youn, Sang Ho Lee, Jung Chul Choi, Jae Eun Jung, Jaehong Kim, Wenchun Qu, Jason Eldrige, Tae-Hun Kim.

**Data curation:** Kiok Kim.

**Funding acquisition:** Kiok Kim, Jaehong Kim.

**Investigation:** Kiok Kim, Sang Ho Lee, Jaehong Kim, Tae-Hun Kim.

**Methodology:** Kiok Kim, Yousuk Youn, Jung Chul Choi, Jae Eun Jung, Jaehong Kim, Wenchun Qu, Jason Eldrige, Tae-Hun Kim.

**Project administration:** Kiok Kim.

**Writing – original draft:** Tae-Hun Kim.

**Writing – review & editing:** Tae-Hun Kim.
